# Supplementary material for: Modeling glioblastoma heterogeneity as a dynamic network of cell states
Source: Mol Syst Biol. 2021 Sep 16;17(9):e10105. doi: 10.15252/msb.202010105 (PMC8444284; doi:10.15252/msb.202010105)
Supplement: Supplementary file 5 — Source Data for Figure 3 [file MSB-17-e10105-s001.zip › Figure3A_sourcedata/GSEA_3065/nosigdb_state5.GseaPreranked.1622718435914/IVY_CTMVP.html]

Details for gene set IVY\_CTMVP[GSEA]

|  || Dataset | state5 |
| Phenotype | NoPhenotypeAvailable |
| Upregulated in class | na\_pos |
| GeneSet | IVY\_CTMVP |
| Enrichment Score (ES) | 0.59599274 |
| Normalized Enrichment Score (NES) | 2.1201966 |
| Nominal p-value | 0.0 |
| FDR q-value | 2.4375001E-4 |
| FWER p-Value | 0.001 |
Table: GSEA Results Summary

  

Fig 1: Enrichment plot: IVY\_CTMVP      
 Profile of the Running ES Score & Positions of GeneSet Members on the Rank Ordered List

  

| PROBE | GENE SYMBOL | GENE\_TITLE | RANK IN GENE LIST | RANK METRIC SCORE | RUNNING ES | CORE ENRICHMENT || 1 | COL4A1 |  |  | 54 | 0.634 | 0.0613 | Yes |
| 2 | COL4A2 |  |  | 93 | 0.540 | 0.1145 | Yes |
| 3 | ITGB1 |  |  | 157 | 0.477 | 0.1581 | Yes |
| 4 | MCAM |  |  | 174 | 0.460 | 0.2052 | Yes |
| 5 | CTGF |  |  | 199 | 0.440 | 0.2493 | Yes |
| 6 | COL18A1 |  |  | 262 | 0.402 | 0.2851 | Yes |
| 7 | LAMC1 |  |  | 382 | 0.348 | 0.3088 | Yes |
| 8 | COL5A2 |  |  | 407 | 0.337 | 0.3420 | Yes |
| 9 | PXDN |  |  | 415 | 0.335 | 0.3769 | Yes |
| 10 | IGFBP7 |  |  | 424 | 0.333 | 0.4114 | Yes |
| 11 | SERPINH1 |  |  | 460 | 0.322 | 0.4418 | Yes |
| 12 | STC1 |  |  | 530 | 0.303 | 0.4662 | Yes |
| 13 | F2R |  |  | 568 | 0.293 | 0.4932 | Yes |
| 14 | LAMB1 |  |  | 619 | 0.281 | 0.5175 | Yes |
| 15 | PCOLCE |  |  | 773 | 0.247 | 0.5266 | Yes |
| 16 | PRSS23 |  |  | 831 | 0.235 | 0.5452 | Yes |
| 17 | GPX8 |  |  | 874 | 0.229 | 0.5649 | Yes |
| 18 | LAMA4 |  |  | 931 | 0.220 | 0.5820 | Yes |
| 19 | FSTL1 |  |  | 1086 | 0.200 | 0.5861 | Yes |
| 20 | TGFBI |  |  | 1177 | 0.188 | 0.5960 | Yes |
| 21 | FN1 |  |  | 1342 | 0.167 | 0.5954 | No |
| 22 | KDELR3 |  |  | 1926 | 0.117 | 0.5426 | No |
| 23 | FRZB |  |  | 1970 | 0.114 | 0.5499 | No |
| 24 | OLFML2A |  |  | 2094 | 0.106 | 0.5474 | No |
| 25 | KCNJ2 |  |  | 2175 | 0.100 | 0.5490 | No |
| 26 | CCDC102B |  |  | 2379 | 0.087 | 0.5356 | No |
| 27 | STEAP1B |  |  | 2769 | 0.064 | 0.4989 | No |
| 28 | ECM1 |  |  | 2809 | 0.062 | 0.5012 | No |
| 29 | TM4SF1 |  |  | 3045 | 0.050 | 0.4802 | No |
| 30 | NID1 |  |  | 3069 | 0.048 | 0.4828 | No |
| 31 | UACA |  |  | 3107 | 0.046 | 0.4836 | No |
| 32 | S1PR3 |  |  | 3308 | 0.036 | 0.4650 | No |
| 33 | THBS1 |  |  | 3755 | 0.017 | 0.4170 | No |
| 34 | SVIL |  |  | 4953 | -0.031 | 0.2864 | No |
| 35 | FILIP1L |  |  | 5096 | -0.037 | 0.2745 | No |
| 36 | TGFBR2 |  |  | 5140 | -0.038 | 0.2737 | No |
| 37 | ARHGAP18 |  |  | 5339 | -0.046 | 0.2565 | No |
| 38 | PHACTR2 |  |  | 5613 | -0.058 | 0.2321 | No |
| 39 | TPM2 |  |  | 5660 | -0.060 | 0.2334 | No |
| 40 | PRDM1 |  |  | 5742 | -0.063 | 0.2311 | No |
| 41 | LBH |  |  | 6577 | -0.107 | 0.1491 | No |
| 42 | TAGLN |  |  | 6733 | -0.116 | 0.1441 | No |
| 43 | ARHGAP29 |  |  | 7007 | -0.138 | 0.1282 | No |
| 44 | PDLIM1 |  |  | 7628 | -0.208 | 0.0809 | No |
| 45 | GNG11 |  |  | 8815 | -0.665 | 0.0189 | No |
Table: GSEA details [plain text format]

  

Fig 2: IVY\_CTMVP: Random ES distribution      
 Gene set null distribution of ES for **IVY\_CTMVP**
